# MALDI Mass Spectrometry Imaging: A Novel Tool for the Identification and Classification of Amyloidosis

**DOI:** 10.1002/pmic.201700236

**Published:** 2017-11-21

**Authors:** Martin Winter, Andreas Tholey, Arnt Kristen, Christoph Röcken

**Affiliations:** ^1^ Department of Pathology Christian‐Albrechts‐University Kiel Germany; ^2^ Systematic Proteome Research & Bioanalytics Institute of Experimental Medicine Christian‐Albrechts‐University Kiel Germany; ^3^ Department of Cardiology, Angiology, and Respiratory Medicine University of Heidelberg Heidelberg Germany

**Keywords:** amyloidosis, formalin‐fixed and paraffin‐embedded, ion mobility separation, MALDI MS imaging

## Abstract

Amyloidosis is a group of diseases caused by extracellular accumulation of fibrillar polypeptide aggregates. So far, diagnosis is performed by Congo red staining of tissue sections in combination with polarization microscopy. Subsequent identification of the causative protein by immunohistochemistry harbors some difficulties regarding sensitivity and specificity. Mass spectrometry based approaches have been demonstrated to constitute a reliable method to supplement typing of amyloidosis, but still depend on Congo red staining. In the present study, we used matrix‐assisted laser desorption/ionization mass spectrometry imaging coupled with ion mobility separation (MALDI‐IMS MSI) to investigate amyloid deposits in formalin‐fixed and paraffin‐embedded tissue samples. Utilizing a novel peptide filter method, we found a universal peptide signature for amyloidoses. Furthermore, differences in the peptide composition of ALλ and ATTR amyloid were revealed and used to build a reliable classification model. Integrating the peptide filter in MALDI‐IMS MSI analysis, we developed a bioinformatics workflow facilitating the identification and classification of amyloidosis in a less time and sample‐consuming experimental setup. Our findings demonstrate also the feasibility to investigate the amyloid's protein composition, thus paving the way to establish classification models for the diverse types of amyloidoses and to shed further light on the complex process of amyloidogenesis.

## 1 Introduction

**Figure 1:**
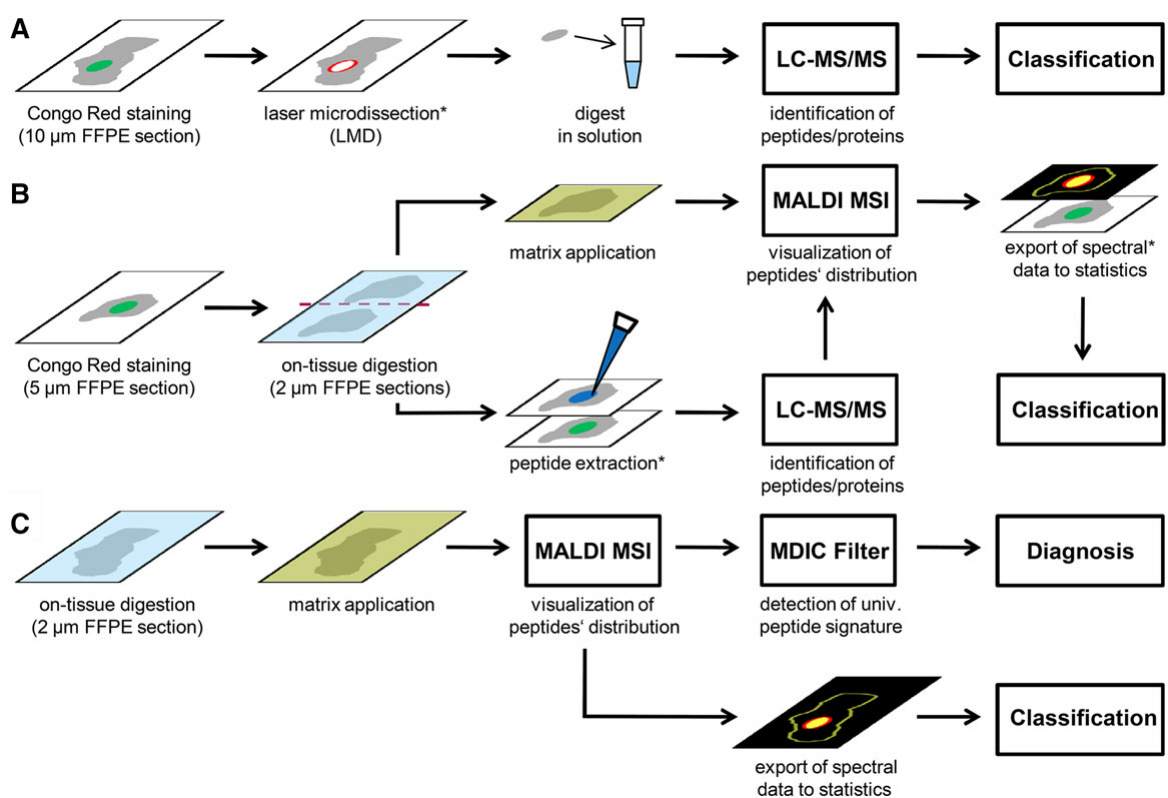
Comparison of the three different mass spectrometry‐based approaches for the identification and classification of amyloidosis: A) Congo red staining followed by laser microdissection coupled with LC‐MS/MS, B) Congo red staining followed by parallel analysis of two consecutive tissue sections by LC‐MS/MS and MALDI MSI, C) MALDI MSI independent from Congo red staining as presented in this study. *Steps that require guidance of the Congo red staining.

Amyloidoses define a large group of diseases caused by extracellular accumulation of misfolded, insoluble polypeptide aggregates, which are oriented in a β‐pleated sheet structure assembling into large fibrillar structures. Amyloid deposits show a characteristic green birefringence in polarized light after staining with Congo red.^1^, ^2^, ^3^ To date, 35 different peptides and proteins are known to form amyloid fibrils.^4^ Since the patient's prognosis and treatment depend on the amyloid type, accurate diagnosis and classification is of paramount importance. In clinical routine tissue‐based diagnosis of amyloidosis is carried out by polarization microscopic examination of formalin‐fixed and paraffin‐embedded (FFPE) tissue sections after Congo red staining. Subsequently, immunostaining on serial sections is most commonly used to identify the amyloid protein.^5^, ^6^ However, unambiguous classification by immunohistochemistry (IHC) can be compromised by background staining due to serum contamination, steric hindrance due to altered 3D confirmation, lack of specific antibodies, and partial or complete proteolytic truncation of the epitope sequence.^7^, ^8^, ^9^, ^10^, ^11^, ^12^ Therefore, additional techniques are required to support the identification of the causative protein. Recently, laser microdissection (LMD) coupled with LC–MS/MS was demonstrated to be a valuable tool for the typing of amyloidosis (Figure 1A).^13^, ^14^, ^15^, ^16^, ^17^ Congo red‐positive regions are dissected by a laser prior to MS analysis to avoid contamination by serum‐derived amyloidogenic proteins of adjacent unaffected tissue. This technique improves the spectra quality significantly and enabled the classification of several amyloidoses with high specificity and sensitivity.^17^, ^18^ It was further shown that the presence of serum amyloid P‐component (SAP) can be used to diagnose amyloidosis in renal biopsies.^16^ Nevertheless, Congo red staining is still required to guide the dissection to obtain pure amyloid samples. A drawback of LC‐MS/MS is the lack of spatial information that hampers a direct assignment of the proteins to amyloid deposits, hence additional measurements need to be done on control samples.

**Significance of the study**

Amyloidoses confine a large group of diseases caused by fibrillar deposits of diverse proteins. Prognosis and therapeutic strategies depend on the nature of the amyloid protein and the underlying disease. Accurate diagnosis and amyloid typing is of paramount importance. Mass spectrometry based proteomics is an upcoming technique in the field of amyloid diagnostics. Here, we developed a straightforward bioinformatics workflow for the tissue‐based analysis of amyloidosis by MALDI‐IMS MSI. In contrast to recent approaches, diagnosis, classification, as well as visualization of the amyloid's distribution is now amenable in a single experiment. Furthermore, this method has also great potential for detailed investigations of the amyloid's protein composition and to unravel the process of tissue remodeling accompanying amyloid formation.

MALDI mass spectrometry imaging (MSI) is a technique providing the detection of both the molecular weight and spatial distribution of biomolecules.^19^, ^20^, ^21^ It has the potential to display colocalized peptides that constitutes an important prerequisite for the identification of proteins and their assignment to histoanatomical structures and pathologies, for example, amyloid.^22^ In a more recent study, MALDI MSI was used to visualize the morphological distribution of amyloidogenic as well as amyloid‐associated proteins after identification by LC‐MS/MS (Figure 1B).^23^ To assign the proteins to amyloid deposits, the peptide images were compared with a Congo red stained serial section. The latter was also used to guide the peptide extraction prior to LC‐MS/MS analysis on a third serial section after on‐tissue digestion. Furthermore, it was demonstrated that MALDI MSI can be used for typing of AA‐ and ALκ amyloidosis in renal FFPE biopsies. For that, spectral data of congophilic regions were selected in the MS image data and exported to build a classification model based on a support vector machine (SVM) algorithm.^24^

In this study, we used MALDI MSI coupled with ion mobility separation (IMS) for the analysis of amyloid‐laden FFPE tissue sections. As protein composition in tissues is highly complex, effects like ion suppression or spectral interferences can hamper unambiguous peptide and protein identification by classical MALDI MSI. The use of IMS allows to separate peptides according to their mobility in the gas phase after ionization, thus providing a tool to reduce the latter effect and therefore to improve the identification and image quality.^25^, ^26^, ^27^, ^28^ Combining the features of both technologies, we developed a peptide filter method enabling a straightforward bioinformatics workflow for the identification and classification of amyloidosis by MALDI‐IMS MSI independent from Congo red staining (Figure 1C).

## 2 Materials and Methods

### 2.1 Patients

Formalin‐fixed and paraffin‐embedded (FFPE) tissue samples from 113 patients were retrieved from the Amyloid Registry of the Christian‐Albrechts‐University of Kiel. For the development of the peptide filter, 16 cases including six different types of amyloid and five different organs were analyzed: brain (ALλ, *n* = 1), heart (AA, *n* = 1; ALλ, *n* = 3; ATTR, *n* = 3), liver (AA, *n* = 1; AApoAI, *n* = 2), kidney (AA, *n* = 2; AFib, *n* = 1; ALys, *n* = 1), and spleen (AA, *n* = 1). The validation cohort consisted of 97 heart tissue samples including 66 amyloid cases (ALλ, *n* = 32; ATTR, *n* = 34) and 31 negative control cases. Identification and classification of amyloid was done prior to this study by Congo red staining analyzed with polarization microscopy and IHC as described in detail elsewhere.^5^, ^6^, ^29^, ^30^ Ethical approval was obtained from the local ethical review board (D 581/15‐585/15). All patient data were pseudonymized before study inclusion.

### 2.2 Materials

Modified sequence grade trypsin was purchased from Promega (Mannheim, Germany) and α‐cyano‐4‐hydroxycinnamic acid (CHCA) from LaserBio Labs (Sophia‐Antipolis Cedex, France). Double‐distilled water (DDW) was obtained from Carl Roth (Karlsruhe, Germany) and xylene from BüFa (Lübeck, Germany). Acetonitrile and ethanol were purchased from Merck (Darmstadt, Germany) and trifluoroacetic acid (TFA), ammonium bicarbonate, octyl‐α/β‐glucoside (OcGlc), red phosphorous, and acetone from Sigma‐Aldrich (Steinheim, Germany).

### 2.3 Sample Preparation

Two micrometer thick tissue sections were cut using a microtome (Leica Biosystems, Nussloch, Germany) and mounted onto histological glass slides (SuperFrost Plus, Menzel‐Gläser). After drying the FFPE tissue sections at 54 °C overnight, paraffin was removed by immersing the sections in xylene (2 × 15 min) followed by rehydration in a series of different graded ethanol solutions (99, 70, and 50% each 2 × 2 min) and double distilled water (DDW, 2 × 1 min). Antigen retrieval was performed in a citric acid buffer (10 mM, pH 6.0) at 100 °C for 30 min utilizing a pressure cooker (Pascal S2800, DakoCytomation California, Inc., USA). The sections were cooled down in a water bath at 10 °C for 15 min, rinsed ten times with DDW and dried in a desiccator at ambient temperature under a vacuum of −800 mBar for at least 15 min. On‐tissue digestion was carried out by spraying ten layers of a trypsin solution (0.05 μg/μL in 50 mM NH_4_HCO_3_ with water/10% ACN:OcGlc, 99.5:0.5, v/v, pH 8.1) with a constant flow rate (10 μL/min) onto the tissue section utilizing the SunCollect Micro Fraction Collector/MALDI Spotter (SunChrom, Friedrichsdorf, Germany) followed by incubation for 2 h at 37 °C in a humid environment using an in‐house incubation chamber. A CHCA matrix solution (5 mg/mL in water/0.2 % TFA:ACN, 50:50, v/v) was applied over eight layers with an increasing flow rate for the first four layers (first at 20 μL/min, second at 30 μL/min, third at 40 μL/min and fourth to eighth at 60 μL/min) resulting in a homogeneous crystallization.

### 2.4 MALDI‐IMS MS Imaging

Imaging experiments were conducted using the MALDI SYNAPT G2‐S (Waters Corporation, Manchester, UK) equipped with a 1 kHz Nd:YAG laser operating in positive ion V‐mode. External calibration of the mass spectrometer was carried out using signals of red phosphorous. Imaging measurements were performed for all cases with IMS in the mass range from 700 to 2000 *m*/*z* at a spatial resolution of 200 μm and 1000 laser shots (1 s) per position. The IMS parameters trap bias direct current (DC), wave velocity (WV), and transfer wave velocity (TWV) were set at 70 V, 350 m/s, and 175 m/s, respectively. Regions for acquisitions were defined with the High Definition Imaging software (HDI) (v1.3.5, Waters) on imported digital scans (Epson Perfection 1640SU) of the tissue sections. Peptide images were generated with the Apex3D algorithm (*m*/*z* window: 0.1 Da, intensity threshold: ten counts; drift window: five bins; IMS peak width: two to ten bins) for the 3000 most intense signals and visualized with the HDI software. The spectral data were normalized against the base peak and recalibrated with an external lock mass (CHCA matrix cluster signal at *m*/*z* 825.101). For the identification of 19 tryptic peptides derived from apolipoprotein AI (ApoAI), apolipoprotein E (ApoE), serum amyloid A (SAA), serum amyloid P component (SAP), transthyretin (TTR), and vitronectin (VTN), the processed imaging data were filtered by mass accuracy, drift time, and image correlation coefficient with tolerances set at 30 ppm, 2.5 bin, and 0.75–1.00, respectively. The peptide image of *m*/*z* 968.55 (ApoE) was used as reference image to determine the Pearson correlation coefficient (*R*)^31^ for the other peptide images utilizing the HDI software. Peptide masses fulfilling all three criteria were considered as detected and identified.

### 2.5 On‐Tissue MALDI MS/MS Imaging

For MS/MS measurements tissue areas covered mostly by amyloid were defined for each peptide by using the HDI software. The precursor masses were selected manually. Peptide fragmentation was conducted in the transfer cell of the TriWave region^32^ and optimized according to the precursor mass. For MS/MS acquisitions without IMS, a 20 V collision energy ramp was utilized and for the IMS mode a fixed collision energy. For the latter mode the TWV was adjusted to 450 m/s. The obtained fragment spectra were processed with the MassLynx software (Waters Corporation), including recalibration with an external lock mass (CHCA matrix‐cluster signal at *m*/*z* 825.101) and deisotoping with the MaxEnt 3 algorithm (maximum molecular mass: precursor mass +20 Da, ensemble members: 1, iterations: 100, intensity threshold: 0.5%). Database searches of the processed spectra were performed using the Proteome Discoverer software package (v1.4.0.288, Thermo Fischer Scientific, Germany) applying the MASCOT (v2.2.07) and SequestHT algorithms. The spectra were searched against a protein fasta database of human nonredundant proteins (20 183 entries, reviewed, downloaded from UniProt, April 07, 2017). The threshold for the estimated false discovery rate was set to 5%. The parent and fragment ion tolerances were set at 25 ppm and ±0.2 Da, respectively. Enzyme specificity was set as trypsin with a maximum of one missed cleavage. All mass spectrometry raw data have been submitted to ProteomeXchange^33^ and are accessible via the identifier PXD005960.

### 2.6 Statistics

All imaging data were reprocessed as described above but with an additional target list consisting of the 19 theoretical peptide masses from ApoAI, ApoE, SAA, SAP, TTR, and VTN as well as their specific drift times. Spectral data of regions showing high signal intensities of the peptide mass at *m*/*z* 968.55 were normalized against the total ion current and exported for statistical analysis using the HDI software. Mann–Whitney *U* test was performed on the 19 peptides using SPSS statistics (v20.0, IBM Corporation, Armonk, USA). All *p* values were taken from two‐tailed tests and were considered statistically significant for *p* < 0.05. A Bonferroni correction was applied to account for false discoveries due to multiple testing. Peptide masses with significant differences in signal intensity were used to build an SVM model (kernel function: radial basis function, sigma: 2, C: 10, cross‐validation: fourfold) with the free available statistics software package Perseus (v1.5.5.3, www.perseus-framework.org).

## 3 Results

**Figure 2:**
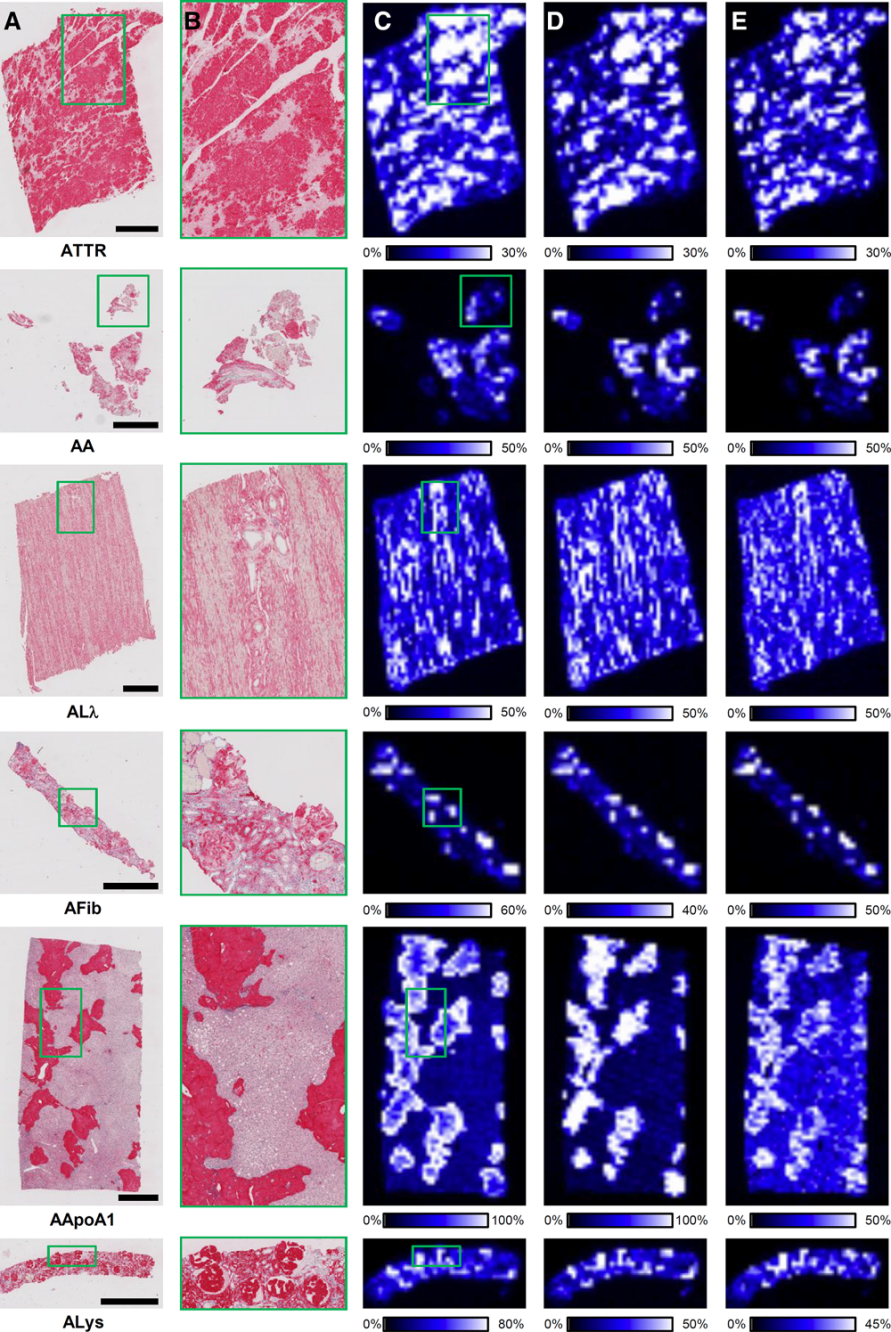
Correlation between immunohistochemistry and MALDI‐IMS MSI analysis of six different amyloid types. Each row displays a panel consisting of immunostaining A), magnification B), images of peptide masses at *m*/*z* 968.55 (ApoE), 1314.68 (VTN), and 1811.89 (SAP) (C–E, respectively). Immunohistochemical staining shows a distribution pattern within the tissue sections similar to the areas with high signal intensities for the peptide masses. Characteristic tissue regions (green rectangle) are magnified for the immunohistochemical staining to visualize the specific enrichment and colocalization of the peptides in amyloid deposits. ATTR: transthyretin‐derived amyloidosis; AA: amyloid A‐derived amyloidosis; ALλ: immunoglobulin λ light chain derived amyloidosis; AFib: fibrinogen‐derived amyloidosis; AApoAI: apolipoprotein AI‐derived amyloidosis; ALys: lysozyme‐derived amyloidosis. The spatial resolution for the peptide images is 200 μm. Scale bar: 2 mM.

In the first set of experiments, we explored a MALDI‐IMS MSI‐based approach for the identification of amyloid deposits in tissue sections obtained from FFPE specimens, which could replace Congo red staining. We investigated 16 amyloid cases including six different types of amyloid and five different histoanatomical locations. For all cases, peptides derived from in silico digest (PeptideMass, www.expasy.org) and recently identified peptide masses^22^, ^23^ formed by tryptic on‐tissue digestion^34^ of ApoE, SAP, and VTN were detected with mass errors below 35 ppm (Table S1, Supporting Information). All three proteins are commonly present in diverse types of amyloid.^22^, ^35^, ^36^ Comparing the peptide images of *m*/*z* 968.55 (ApoE), *m*/*z* 1314.68 (VTN), and *m*/*z* 1811.89 (SAP) with the immunostaining of the corresponding amyloid type demonstrates a specific spatial enrichment and colocalization in amyloid deposits (Figure 2). Colocalization was confirmed by Congo red staining and polarization microscopy of the same tissue sections used for MALDI‐IMS MSI analysis after washing off the matrix with 80% ethanol. Furthermore, peptide masses of the amyloidogenic proteins ApoAI (in silico digest), SAA (identified^23^), and TTR (identified^37^) were detected in their corresponding amyloid cases with mass errors below 25 ppm (Table S1, Supporting Information). Based on these data, we built a target list of 19 peptides belonging to the six proteins. To confirm the identity of the peptides MALDI MS/MS was conducted directly on FFPE tissue sections of three different amyloid cases with AA‐, AApoAI‐ (both liver), and ATTR (heart) amyloidosis. All peptide masses were identified as tryptic and proteotypic peptides in at least one case with a false discovery rate <1% (Table S2, Supporting Information). An example for the fragment spectra is given in Figure S1, Supporting Information. Furthermore, for each peptide mass a specific drift time was obtained due to the IMS.

Subsequently, we designed a peptide filter (MDIC) including the three parameters: (1) mass accuracy (M), (2) drift time (D), and (3) an image correlation coefficient (IC) (Figure S2, Supporting Information). Reference masses were calculated by in silico digests of the six proteins using PeptideMass (www.expasy.org). The mass error was below 30 ppm for 96% (201 of 210) of all detected peptide masses, thus defining the tolerance for M. The peptide image of *m*/*z* 968.55 (ApoE) was used as reference image to determine the peptides’ IC, since this peptide mass was detected for all cases and with good signal intensities. This resulted in a Pearson correlation coefficient of at least 0.75 for 95% (199 of 210) of the detected peptide masses, thus defining the minimum threshold of IC. The reference values for the drift time parameter were determined by averaging the drift times of the corresponding peptide masses fulfilling the mass accuracy and correlation criteria. Comparing the mean values with the measured drift times, the drift time error was below 2.5 bin for 97% (203 of 210) of the detected peptide masses, hence defining the tolerance for D. References for the peptide masses and drift times used for the peptide filter are listed in Table S3, Supporting Information.

**Figure 3:**
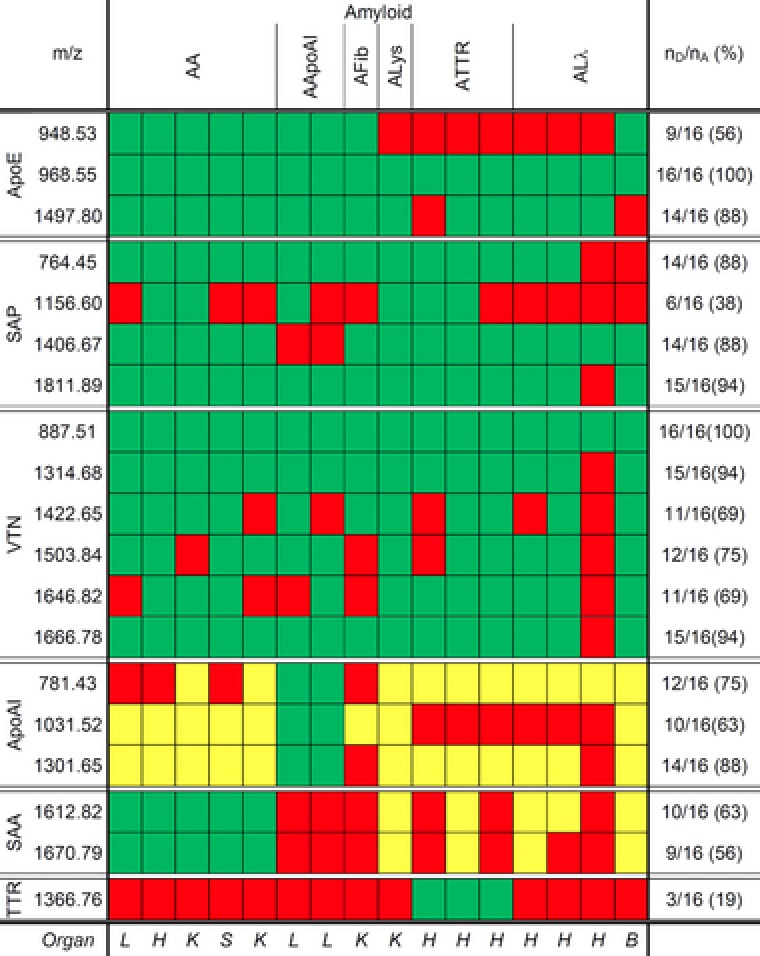
Overview of the 16 different cases with AA‐, AApoAI‐, AFib‐, ALys‐, ATTR‐, and ALλ amyloidosis, respectively, in FFPE tissue sections from brain (B), heart (H), kidney (K), liver (L), or spleen (S). For each case it is shown, which peptides of the common components ApoE, SAP, VTN, and the amyloidogenic proteins ApoAI, SAA, TTR were detected (green) or not detected (red). Peptides of ApoAI and SAA were also detected for noncorresponding amyloid cases (yellow). The detection frequency of each peptide mass was determined by dividing the number of amyloid cases with detection (*n*_D_) by the number of all amyloid cases (*n*_A_).

Figure 3 shows the detected peptides for each amyloid case when applying the MDIC peptide filter on the processed imaging data. For the common components, each *m*/*z* value was detected in at least 14 of 16 cases (88%) except for *m*/*z* 948.53 (56%, ApoE), *m*/*z* 1156.60 (38%, SAP), *m*/*z* 1422.65, *m*/*z* 1503.84, and *m*/*z* 1646.82 (69,75 , and 69%, respectively, VTN). Examining the imaging data for peptide masses originating from amyloidogenic proteins revealed that the peptides of ApoAI and SAA were also detected for other than the corresponding amyloidoses. In contrast, the peptide mass at *m*/*z* 1366.76 of TTR was detected only in ATTR amyloid deposits. Therefore, we considered a variable composition of the peptides belonging to ApoE, SAP, and VTN as the universal peptide signature for amyloid deposits, whereas the peptides of ApoAI, SAA, and TTR can be used as additional indicators. Furthermore, it might be possible to distinguish ALλ from ATTR amyloidosis in heart tissue by the absence or presence of the peptide mass at *m*/*z* 1366.76, respectively.

Next, we performed MALDI‐IMS MSI analysis on an independent validation cohort of 97 heart tissue samples including 32 cases with ALλ amyloidosis, 34 with ATTR amyloidosis, and 31 control cases without amyloid. Applying the peptide filter on the processed imaging data, the peptides were found in 62 of 66 amyloid cases but not in a single case without amyloid (negative control), thereby reaching a sensitivity of 94% and a specificity of 100% for the identification of amyloid deposits. Subsequently, the result was verified by staining all tissue sections analyzed by MALDI‐IMS MSI with Congo red and inspection in polarized light: all amyloid cases were positive and all control cases were negative for amyloid. Furthermore, the combination of the detected peptides varied again for each case, thus resulting in different detection frequencies for each peptide mass (Table 1).

**Table 1:** Detection frequencies of peptides in ALλ and ATTR amyloid cases of the validation cohort and *p* values of the Mann–Whitney *U* test

Protein	*m*/*z*	Amyloid		ALλ‐amyloid		ATTR‐amyloid		ALλ versus ATTR
		*n*_D_/*n*_A_	(%)	*n*_D_/*n*_ALλ_	(%)	*n*_D_/*n*_ATTR_	(%)	*p* Values
ApoE	*948.53*	22/66	33	16/32	50	6/34	18	0.396
	*968.55*	62/66	94	31/32	97	31/34	91	0.562
	*1497.80*	48/66	73	23/32	72	25/34	74	0.448
SAP	*764.45*	29/66	44	8/32	25	21/34	62	<*0.001* a)
	*1156.60*	11/66	17	0/32	0	11/34	32	<*0.001* a)
	*1406.67*	60/66	91	30/32	94	30/34	88	<*0.001* a)
	*1811.89*	51/66	77	20/32	63	31/34	91	<*0.001* a)
VTN	*887.51*	29/66	44	12/32	38	17/34	50	0.203
	*1314.68*	56/66	85	27/32	84	29/34	85	0.503
	*1422.65*	56/66	85	29/32	91	27/34	79	<*0.001* a)
	*1503.84*	17/66	26	10/32	31	7/34	21	0.633
	*1646.82*	36/66	55	18/32	56	18/34	53	0.114
	*1666.78*	37/66	56	20/32	63	17/34	50	0.944
ApoAI	*781.43*	53/66	80	28/32	88	25/34	74	0.688
	*1031.52*	42/66	64	25/32	78	17/34	50	0.967
	*1301.65*	60/66	91	30/32	94	30/34	88	0.853
SAA	*1612.82*	10/66	15	4/32	13	6/34	18	0.146
	*1670.79*	11/66	17	6/32	19	5/34	15	0.843
TTR	*1366.76*	36/66	55	10/32	31	26/34	76	<*0.001* a)

a) Significant differences in signal intensity. Detection frequency for each peptide mass when considering the number of detections (*n*_D_) in all amyloid cases (*n*_A_) as wells as in all ALλ (*n*_ALλ_) and all ATTR cases (*n*_ATTR_) of the validation cohort. The *p* values were determined by conducting a Mann–Whitney *U* test on the exported spectral data of all amyloid cases using a basic significance level of *p* < 0.05 with additional Bonferroni correction. Peptide masses with significant differences in signal intensity were used for the SVM classification model.

Grouping the amyloid cases according to the type of amyloidosis revealed that the peptide mass of TTR was not exclusively detected in tissue samples with ATTR amyloid. Thirteen (41%) specimens with ALλ amyloid also harbored the peptide with *m*/*z* 1366.76 (TTR). However, it was more commonly found in ATTR amyloid as were the peptide masses at *m*/*z* 764.45, *m*/*z* 1156.60, and *m*/*z* 1811.89 (all SAP). To the contrary, the peptide masses at *m*/*z* 948.53 (ApoE) and *m*/*z* 1031.52 (ApoAI) were more prevalent in ALλ amyloid. The results indicated that the two amyloid types might be discriminated by these peptides’ signal intensities. Subsequently, spectral data of regions showing high signal intensities in the peptide image of *m*/*z* 968.55 were selected in all amyloid samples and submitted to a Mann–Whitney *U* test resulting in significantly higher signal intensities of TTR (*m*/*z* 1366.76), VTN (*m*/*z* 1422.65), and all SAP peptides for ATTR amyloid (*p* < 0.001, Table 1).

Finally, based on these six peptide masses, we built an SVM model to classify ALλ and ATTR amyloidosis. After cross‐validation, ATTR was distinguished from ALλ with a sensitivity and specificity of 91.2% (true ATTR: 31, false ALλ: 3) and 93.8 % (true ALλ: 30, false ATTR: 2), respectively. This results in a prediction value of 93.9% for ATTR and 90.9% for ALλ.

## 4 Discussion

Tissue‐based diagnosis of amyloid is carried out by polarization microscopic examination of FFPE tissue sections after Congo red staining. Once the presence of amyloid deposits is confirmed, classification of the amyloid protein is essential to guide the patient's treatment. Currently, IHC is the most commonly utilized method to identify the causative protein, but harbors some difficulties compromising the specificity and sensitivity.

Here, we introduced an approach enabling the diagnosis and classification of amyloid deposits in FFPE tissue sections by MALDI‐IMS MSI analysis. Combining the features of MALDI imaging and the ion mobility separation, we developed a filter method discriminating the detected tryptic peptides by mass accuracy, drift time, and an image correlation coefficient. When performing imaging experiments, we observed relatively high mass errors for the peptides, thus identification by their masses only would be fraught with uncertainty. We demonstrated that it is possible to identify the peptides unambiguously by MS/MS analysis directly on serial sections (Table S2, Supporting Information). However, the performance of on‐tissue MS/MS depends on the signal intensity of the precursor peptide ion and can be compromised by insufficient amount of the corresponding protein at the given tissue position and ion suppression due to the complexity of the sample. Its application is therefore restricted to peptides of abundant proteins or with a high ionization efficiency. Furthermore, implementing this step in the workflow would be time and sample consuming, preventing its application in fast clinical routines. Instead, we used the drift time and the image correlation coefficient as additional parameters to increase the confidence of the peptide identification. The drift time was obtained from the ion mobility separation and is specific for each peptide. The image correlation coefficient takes advantage of the fact that in case of amyloidosis, the peptides of amyloidogenic and amyloid‐associated proteins are colocalized and specifically enriched in the amyloid deposits. This results in peptide images with similar distribution patterns (Figure 2) and hence in high positive Pearson correlation coefficients. As yet, peptide images were compared manually to decide if peptides are colocalized or not, whereby the contrast is often adjusted individually to visualize characteristic distribution patterns within the tissue sample (Figure 2).^23^ Therefore, a manual comparison is time consuming and lacks reproducibility as well as comprehensibility, since the decision is made according to the user's perception only. Introducing an image correlation coefficient overcomes the aforementioned issues. Since its determination is carried out by comparing the peptide images according to a certain software algorithm, this approach is not only faster (a few seconds), but also reproducible. Moreover, colocalization is now quantified and allows for a comprehensible decision based on a numerical value. In summary, discriminating the peptides by all three criteria constitutes a reliable method for the detection and identification of peptides accumulated in amyloid deposits.

Applying the MDIC peptide filter to the imaging data, we found a universal peptide signature with a variable composition of tryptic peptides belonging to the six proteins ApoE, SAP, VTN, ApoAI, SAA, and TTR, which enables the identification of amyloid deposits with high sensitivity and specificity. It is noteworthy that the presence of the peptide mass at *m*/*z* 968.55 (mass error ≤ 30 ppm, drift error ≤ 2.5 bin) derived from ApoE is essential for the application of this peptide filter. Therefore, none of the peptides were detected in all negative control cases and four amyloid cases (one ALλ, three ATTR) of the validation cohort. Since this peptide mass was also found to display the amyloid deposits the best, it can be considered as the Congo red equivalent for MALDI MSI analysis. However, a peptide of ApoE may be an unsuitable marker to separate mature Congo red positive amyloid fibrils from prefibrillar Congo red negative protein aggregates in the early stages of the disease: ApoE can form aggregates with nonfibrillar amyloid proteins.^38^ Furthermore, peptides of TTR and SAP were more commonly found in ATTR amyloid than ALλ amyloid. The peptides’ signal intensities were also found to be significantly higher for ATTR. Interestingly, the observations made for the peptides of SAP were in good agreement with the IHC, since the anti‐SAP‐staining of AL amyloidosis is found to be often weak and spotty (Röcken C, unpublished observation). This result lends further support that amyloidoses have not only characteristic distribution patterns according to the amyloid type and histoanatomical distribution,^39^ but differ also in the composition of their common components.^22^, ^40^ Anyway, based on our findings, we were able to build an SVM model that makes it possible to classify the two most prevalent amyloid types in cardiac amyloidosis^41^ with high sensitivity and specificity.

We present here a novel bioinformatics workflow, which allows the investigation of amyloidoses in a simple way and offers several intriguing advantages: (1) the application of the MDIC peptide filter enables a targeted identification of tryptic peptides in amyloid deposits based on MALDI‐IMS MS imaging data. Therefore, the additional time consuming step of MS/MS analysis is not required. (2) Amyloid can be identified and diagnosed in a preserved histoanatomical context of a tissue section by MALDI MSI based on the detection of a universal peptide signature. Furthermore, the same tissue specimen can be forwarded to additional histochemical studies such as Congo red‐staining. (3) In contrast to LMD‐LC‐MS/MS,^16^ the integrity of the tissue is preserved, thus facilitating to display the amyloid's distribution within the tissue section immediately by the peptide mass at *m*/*z* 968.55. This allows the direct export of the spectral data of amyloid deposits for statistical analysis, without the guidance of a Congo red stained serial section (Figure 1B and C). (4) Due to the fact, that peptides characteristic for amyloid deposits will result in peptide images similar to the image of *m*/*z* 968.55, the image correlation coefficient can be used for an untargeted screening of the imaging data to discover new potential biomarkers of amyloidoses. Once the peptides were identified by MS/MS analysis, they can be added to the peptide filter's target list to increase the confidence of amyloid diagnosis. (5) Retrospective studies of imaging data might reveal them as potential discriminators similar to the peptides of SAP in this study. This will not only allow to build new or improved SVM classification models, but also to investigate the amyloid's proteome to gain further insights into its complex pathogenesis. (6) In a recent study, we revealed that VTN is not homogenously distributed within the amyloid deposits.^22^ Acquiring peptide images with higher spatial resolution, it might be possible to detect heterogenic composition pattern within the amyloid plaques, thus enabling a more detailed examination of the tissue remodeling processes during amyloid accumulation.

In conclusion, we demonstrated the feasibility of MALDI‐IMS MSI to be used as an independent analytical method for the identification and classification of amyloidosis in FFPE tissue sections. MALDI MSI is a very young technique in the field of amyloid diagnostics. Therefore, cohorts including further amyloid types and organs need to be investigated in future studies to extend its application. Introducing the MDIC peptide filter enabled a straightforward bioinformatics workflow for the investigation of the amyloid's protein composition with great potential to discover new biomarkers of amyloidoses. Furthermore, little is known if and how the different components enclosed in amyloid deposits contribute to the formation of amyloid fibrils. Our approach might also help to unravel the complex process of amyloidogenesis, which is an important prerequisite for the development of new therapeutic strategies.

**Abbreviations**
DDW: double‐distilled water
FFPE: formalin‐fixed and paraffin‐embedded
IHC: immunohistochemistry
IMS: ion mobility separation
MSI: mass spectrometry imaging
SAP: serum amyloid P‐component
SVM: support vector machine
VTN: vitronectin

## Conflict of Interest

The authors declare no competing financial interest.

## Supporting information

supporting information
