# Supplementary material for: MALDI Mass Spectrometry Imaging: A Novel Tool for the Identification and Classification of Amyloidosis
Source: Proteomics. 2017 Nov 21;17(22):1700236. doi: 10.1002/pmic.201700236 (PMC5725723; doi:10.1002/pmic.201700236)
Supplement: Supplementary file 1 — supporting information [file PMIC-17-na-s001.docx]

**Supporting Information**

**MALDI Mass Spectrometry Imaging: a novel Tool for the**

**Identification and Classification of Amyloidosis**

Martin Winter^1^, Andreas Tholey^2^, Arnt Kristen^3^, and Christoph Röcken^1^*

^1^Department of Pathology, Christian-Albrechts-University, 24105 Kiel, Germany

^2^Systematic Proteome Research & Bioanalytics, Institute of Experimental Medicine, Christian-Albrechts-University, 24105 Kiel, Germany

^3^Department of Cardiology, Angiology, and Respiratory Medicine, University of Heidelberg, 69120 Heidelberg, Germany

*Tel: +49 431-500-15501, Fax: +49 431-500-15504

E-mail: christoph.roecken@uk-sh.de

**Table of Content**

| **Table 1** | Mass accuracies obtained by MALDI-IMS MSI analysis of 16 different amyloid cases. |
| --- | --- |
| **Table 2** | List of peptides identified by MALDI MS/MS directly on FFPE tissue. |
| **Table 3** | List of peptide masses and drift times used as reference in the MDIC peptide filter. |
| **Figure 1** | Example for the fragment spectra obtained by on-tissue MALDI MS/MS experiments. |
| **Figure 2** | Development of the peptide filter used for the detection and identification of tryptic peptides in amyloid deposits. |


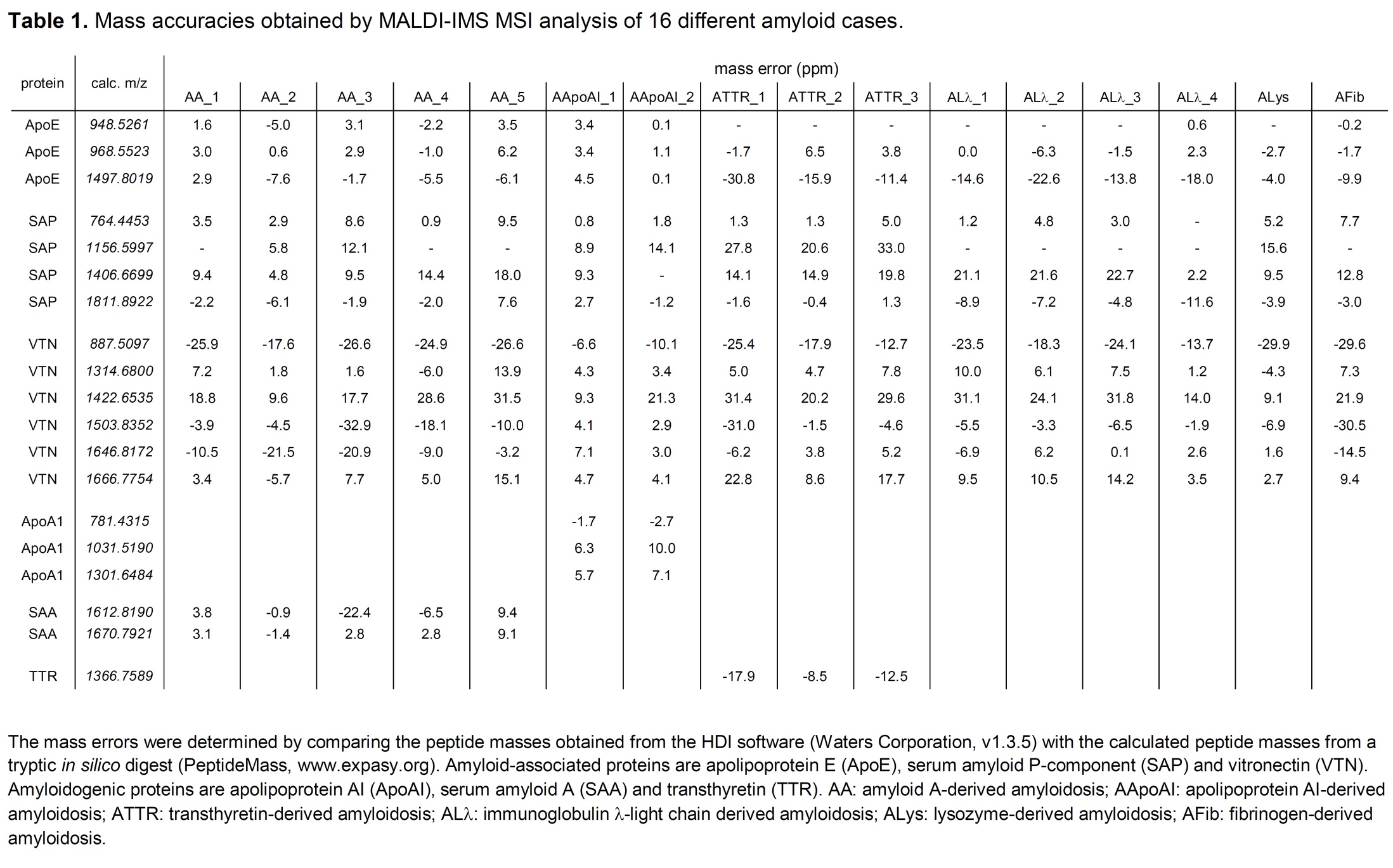


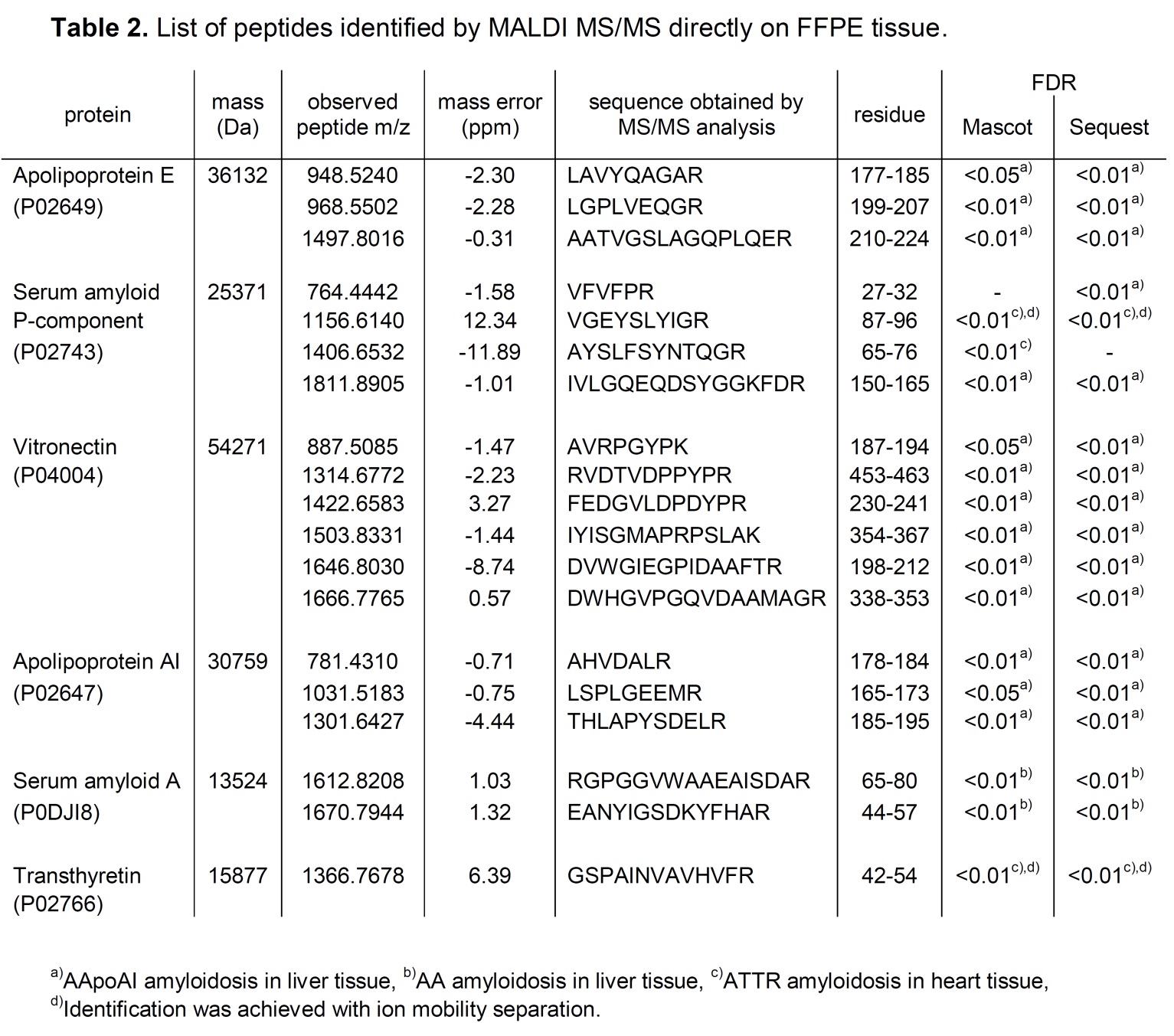


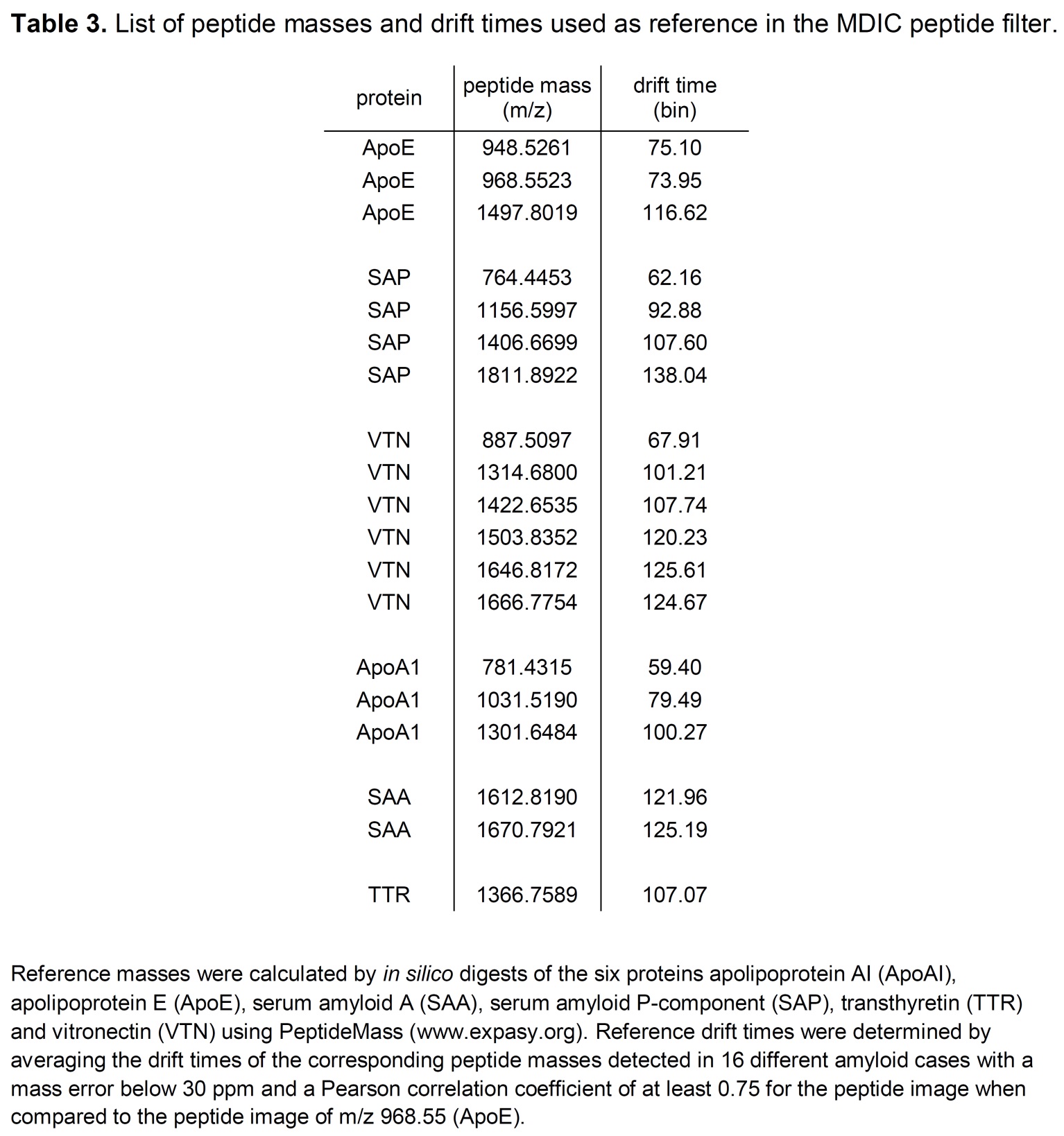


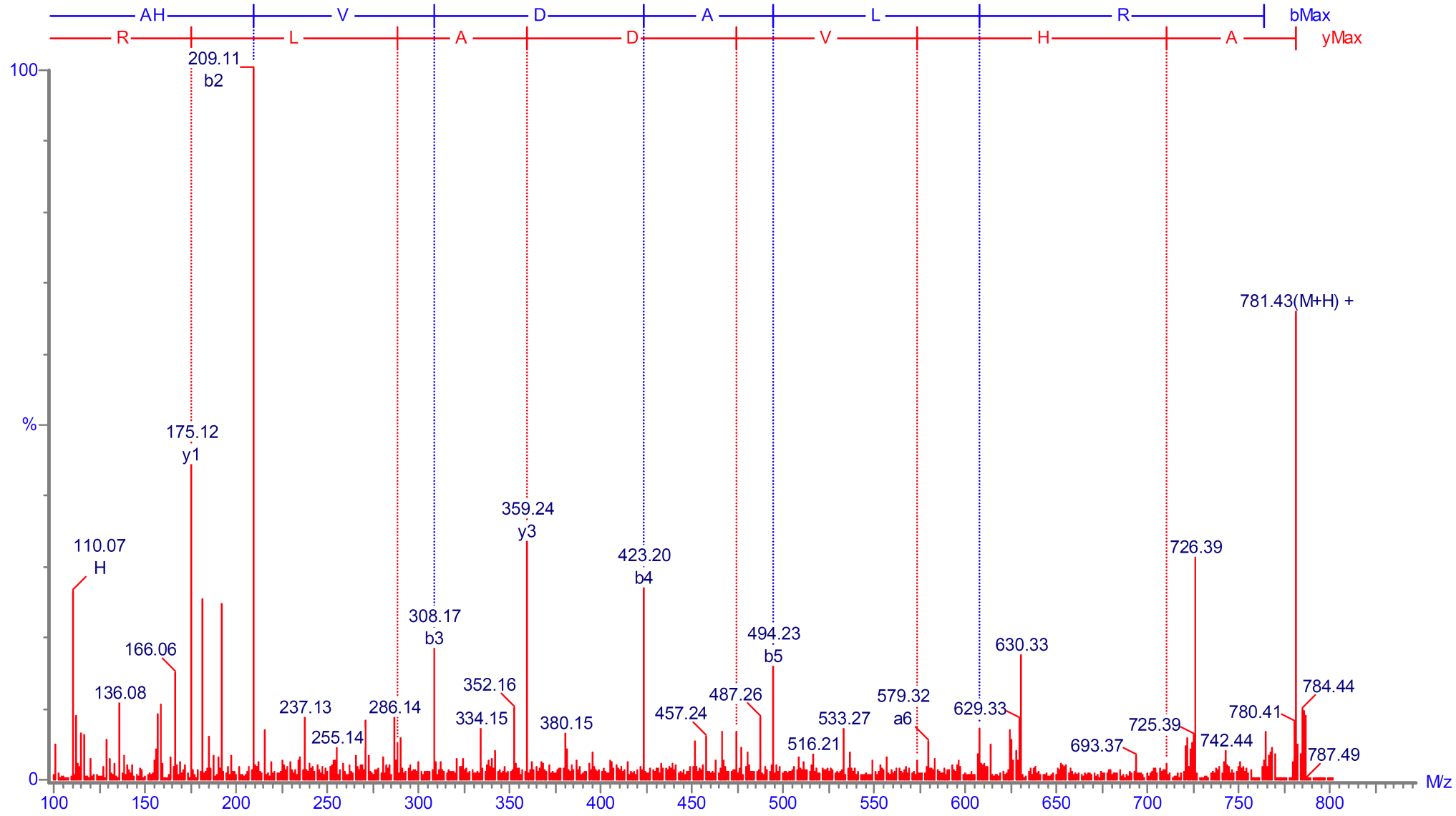


**Figure 1.** Example for the fragment spectra obtained by on-tissue MALDI MS/MS experiments. The spectrum displays the fragmentation pattern of the tryptic peptide at m/z 781.43 identified as ApoAI and was acquired from liver tissue containing AApoAI amyloid. The fragment annotations were made with the interactive MS/MS sequencing tool PepSeqTM (Waters Corporation, Manchester, U.K.).


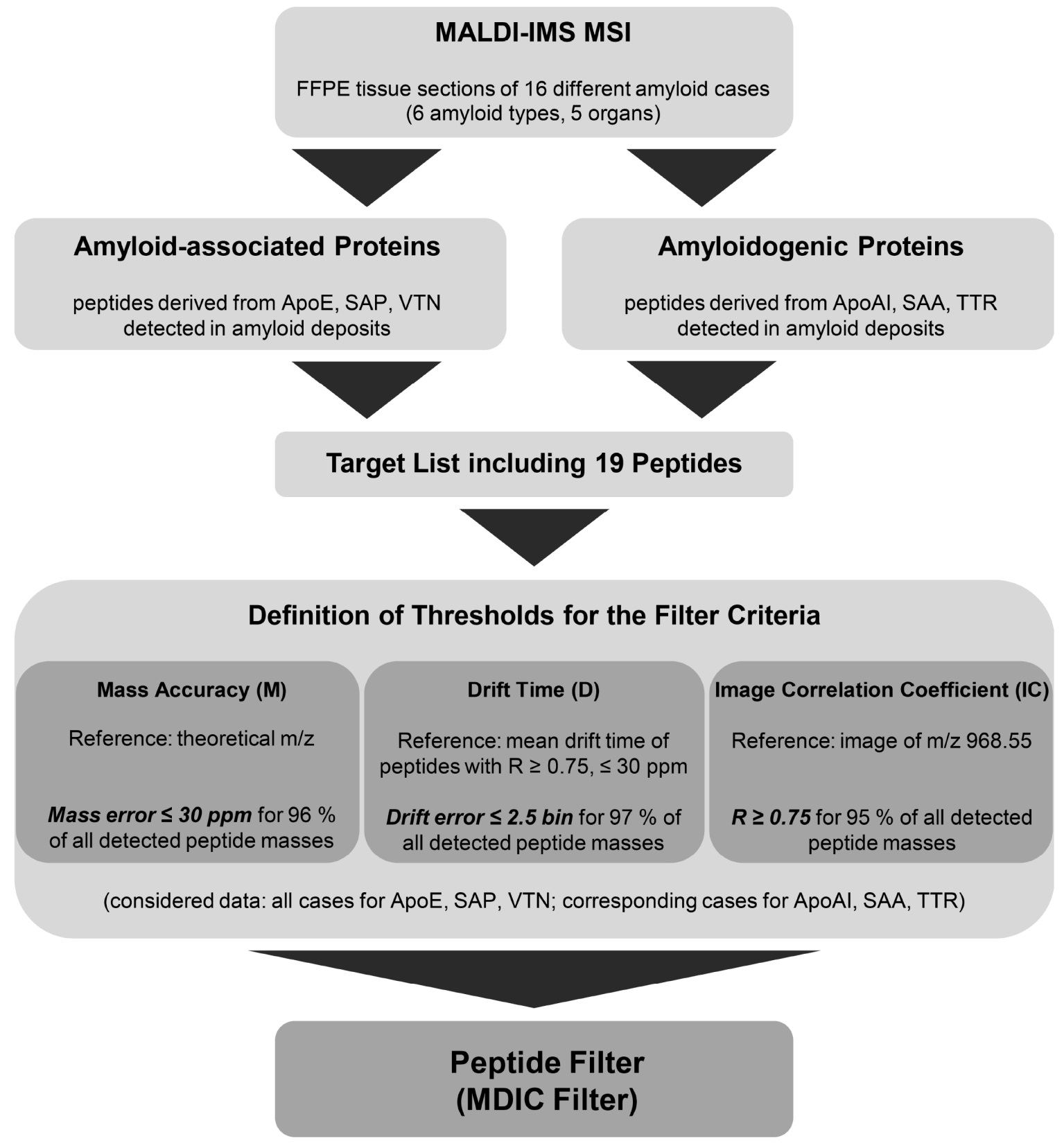


**Figure 2.** Development of the peptide filter used for the detection and identification of

tryptic peptides in amyloid deposits.
